# Invasive Fungal Infections After Intestine Transplantation: Epidemiology and Outcomes

**DOI:** 10.1111/myc.70180

**Published:** 2026-04-28

**Authors:** Mayyadah H. Alabdely, Jessica Lum, Blanca E. Gonzalez, Masato Fujiki, Zachary A. Yetmar

**Affiliations:** ^1^ Department of Infectious Disease Cleveland Clinic Foundation Cleveland Ohio USA; ^2^ Center for Pediatric Infectious Disease, Cleveland Clinic Foundation Cleveland Ohio USA; ^3^ Center for Gut Rehabilitation and Transplantation, Cleveland Clinic Foundation Cleveland Ohio USA

**Keywords:** allograft loss, IFI, intestinal transplant, outcome, risk factors

## Abstract

**Background:**

Invasive fungal infections (IFIs) represent a major complication after intestine transplantation, with reported incidence rates between 40% and 49%. These infections are associated with high morbidity and allograft loss. This study evaluates the impact of post‐transplant IFIs on graft outcomes in intestine transplant recipients.

**Methods:**

We conducted a retrospective cohort study of 152 patients who underwent intestine transplantation from 2008 to 2022. The primary outcome was IFI, defined as proven or probable by EORTC/MSGERC criteria. The secondary outcome was a composite of allograft failure or death. Analyses were conducted using multivariable Cox proportional hazards models, with non‐baseline variables incorporated as time‐dependent exposures.

**Results:**

Among 152 intestine transplant recipients, 56 (36.8%) developed post‐transplant IFI. Median time to IFI was 83.5 days (IQR 19.5–444.2), with *Candida* infections occurring early and non‐*Candida* infections occurring later. *Candida* species accounted for 73% of IFIs, most commonly *C. glabrata* , *C. albicans* , and *C. parapsilosis* , with 29.3% showing fluconazole resistance. Most infections were intra‐abdominal and bloodstream. Redo transplantation (HR 2.44, 95% CI 1.20–4.95; *p* = 0.014), anastomotic leak (HR 2.23, 95% CI 1.02–4.90; *p* = 0.045), and augmented immunosuppression (HR 4.73, 95% CI 1.94–11.53; *p* < 0.001) were independent predictors of Candida IFI. IFI was associated with a markedly increased risk of allograft loss or death (HR 3.67, 95% CI 2.30–5.83; *p* < 0.001).

**Conclusions:**

Post‐transplant IFIs are common and associated with allograft loss or mortality in intestine transplant recipients. Early recognition and aggressive management of IFIs remain critical to improving transplant outcomes.

## 1 Introduction

Invasive fungal infections (IFIs) pose a significant threat to patients who undergo intestine transplantation, with incidence rates reported between 40% and 49%—the highest among all solid organ transplant recipients [1, 2]. *Candida* species are the predominant pathogens, followed by *Aspergillus* species and other moulds like *Mucorales*. In the paediatric population, *Candida* infections are especially prevalent, commonly presenting as candidemia or intra‐abdominal infections [3]. The latter typically arises within the first month post‐transplant, whereas bloodstream infections tend to occur later, often after six months [2].

Several risk factors contribute to the high rate of IFIs in these patients. These include prolonged and intense immunosuppression, compromised mucosal integrity due to surgery or complications like anastomotic leaks and ischemia, central venous catheters, extended use of broad‐spectrum antibiotics, and episodes of rejection necessitating increased immunosuppression [2, 4]. The gastrointestinal tract serves as a major reservoir for *Candida*, with infections arising from both internal colonisation and external sources [3].

Despite recognition of these risks, the overall epidemiology and outcomes of IFI after intestine transplantation remain poorly defined [3, 5, 6]. This lack of comprehensive data hampers the development of standardised prevention and management strategies. Additional studies are therefore needed to better characterise the burden, risk factors, and consequences of IFI in this uniquely vulnerable population. We sought to evaluate our centre's cohort of intestine transplant recipients to assess the risk of post‐transplant IFI and its effect on long‐term transplant outcomes.

## 2 Methods

### 2.1 Study Design and Population

We performed a retrospective cohort study of adults and paediatric patients who underwent intestine transplantation at our institution between 1 January 2008 and 31 December 2022. **Intestine transplantation included** isolated intestine, liver–intestine, modified multivisceral, and multivisceral transplants [7]. Patients were excluded if they died or experienced intestinal allograft failure within 2 days of transplantation. Eligible patients were identified through our institutional transplant registry. Demographic, clinical, transplant‐related, and outcome data were manually abstracted from the electronic medical record. This study was reviewed by our Institutional Review Board and granted an exemption from informed consent (IRB #24–136). No written consent has been obtained from the patients, as there is no patient‐identifiable data included.

The primary outcome was IFI after intestinal transplantation. IFI was defined using the proven or probable categorisations based on EORTC/MSGERC criteria [8]. The secondary outcome was a composite of allograft failure and all‐cause mortality anytime during post‐transplant follow‐up. Allograft failure was defined as allograft enterectomy. Augmented immunosuppression was defined as the use of either intravenous corticosteroids, Alemtuzumab, or antithymocyte globulin (ATG), typically administered for the treatment of rejection. All outcomes were assessed over the entire available follow‐up period. Pretransplant IFI was defined as IFI reported at any time prior to transplant.

Intra‐abdominal candidiasis was adjudicated based on isolation of *Candida* from normally sterile intra‐abdominal specimens or from drains placed within ≤ 24 h in patients with clinical evidence of infection. In contrast, cultures obtained from non‐sterile sources or from drains in place for > 24 h without supportive clinical findings were consideredcolonisation.

### 2.2 Transplantation Protocols

Perioperative antibacterial prophylaxis typically consisted of vancomycin, piperacillin–tazobactam, and metronidazole, administered for 7 days following transplantation. In patients with documented beta‐lactam allergy, piperacillin–tazobactam was replaced with aztreonam. Metronidazole was included to ensure optimal anaerobic coverage, particularly for *Bacteroides* species. All transplant candidates underwent an infectious disease evaluation prior to surgery, with perioperative regimens modified when prior microbiologic data or infection history warranted. Routine screening for resistant organisms was not performed.

Universal antifungal prophylaxis was administered with micafungin, except between 2015 and 2017 when amphotericin B lipid complex was used. Antifungal prophylaxis was continued for the entirety of the index post‐transplanthospitalisation. *Pneumocystis* prophylaxis was prescribed indefinitely, preferentially with trimethoprim–sulfamethoxazole unless contraindicated, in which case an alternative agent was selected. Antiviral prophylaxis with ganciclovir or valganciclovir was continued for at least 6 months post‐transplant for all patients.

Induction immunosuppression initially consisted of ATG until 2012, after which alemtuzumab became the standard. ATG use after 2012 was limited to paediatric recipients or adults at increased risk for infection, with prior malignancy, or with substantial bleeding risk. Maintenance immunosuppression generally included tacrolimus and corticosteroids, with the latter tapered early to a lower maintenance dose.

### 2.3 Statistical Analysis

Continuous variables were summarised as median with interquartile range (IQR), and categorical variables as number with percentage. Continuous and categorical variables were compared between groups using the Wilcoxon rank‐sum test and Fisher's exact test, respectively. Time‐to‐event outcomes were analysed using the Kaplan–Meier method, with differences assessed by the log‐rank test. Outcomes were then assessed by multivariable Cox regression. Due to suspected differences in risk and a low number of non‐*Candida* IFI events, *Candida* IFI was only evaluated in regression analysis. Variables were selected for the models a priori based on prior studies, suspected confounding, and biologic plausibility of association. Any predictor variables that were not known at baseline, such as acute rejection or IFI, were analysed as time‐dependent exposures. A *p*‐value of less than 0.05 was considered statistically significant. Statistical analysis was performed using R 4.2.3 (R Foundation for Statistical Computing).

## 3 Results

### 3.1 Patient Cohort

A total of 137 patients who underwent 152 intestinal transplant surgeries were included in this study after excluding 3 patients who died or experienced allograft failure within 2 days of transplantation. The cohort had a mean age of 38.1 years, was 46.1% male, and had a mean body mass index (BMI) of 23.2 kg/m^2^ (Table 1). 53 recipients (36%) had a documented IFI before transplantation, the majority of which were caused by *Candida* species (*n* = 49), followed by *Aspergillus* species (*n* = 2), *Histoplasma* (*n* = 1), and *Cryptococcus* (*n* = 1).

**TABLE 1:** Baseline characteristics grouped by occurrence of invasive fungal infection.

Variable	Entire cohort (*N* = 152)	No Post‐Transplant IFI (*n* = 96)	Post‐Transplant IFI (*n* = 56)	*p*‐value
Demographics				
Age at transplantation, years	38.3 ± 17.4	38.7 ± 17.5	37.5 ± 17.3	0.689
Sex				1.000
Female	82 (53.9%)	52 (54.2%)	30 (53.6%)	
Male	70 (46.1%)	44 (45.8%)	26 (46.4%)	
Body mass index, kg/m^2^	23.2 ± 4.4	23.5 ± 4.3	22.8 ± 4.7	0.375
Race				0.464
Asian	3 (2.0%)	1 (1.0%)	2 (3.6%)	
Black or African American	11 (7.2%)	6 (6.2%)	3 (5.4%)	
White	114 (75.0%)	74 (77.1%)	40 (71.4%)	
Other	24 (15.8%)	13 (13.5%)	11 (19.6%)	
Charlson Comorbidity Index	0.91 ± 1.5	0.67 ± 1.2	1.32 ± 2.0	0.012
Pre‐transplant IFI History	53 (34.9%)	33 (34.4%)	20 (35.7%)	1.000
*Candida* species	49 (32.2%)	31 (32.3%)	18 (32.1%)	0.652
Transplant Characteristics				
Transplant Type				0.019
Isolated Intestine	98 (64.5%)	67 (69.8%)	31 (55.4%)	
Liver‐Intestine	12 (7.9%)	3 (3.1%)	9 (16.1%)	
Modified multivisceral	15 (9.9%)	11 (11.5%)	4 (7.1%)	
Multivisceral	27 (17.8%)	15 (15.6%)	12 (21.4%)	
Redo Transplant	23 (15.1%)	9 (9.4%)	14 (25.0%)	0.018
Operative Time, minutes	679.5 ± 132	663.5 ± 120	706.9 ± 147	0.071
Immunosuppression & Prophylaxis				
Induction Agent				0.018
Alemtuzumab	106 (69.7%)	73 (76.0%)	33 (58.9%)	
Antithymocyte globulin	25 (16.4%)	16 (16.7%)	9 (16.1%)	
None/Other	21 (13.8%)	7 (7.3%)	14 (25.0%)	
Antifungal Prophylaxis				0.738
Amphotericin B lipid complex	36 (23.7%)	23 (24.0%)	13 (23.2%)	
Micafungin	115 (75.7%)	72 (75.0%)	43 (76.8%)	
Prophylaxis Duration, days	28.4 ± 22.4	26.4 ± 22.6	31.8 ± 21.9	0.161
Surgical Complications				
Chyle Leak	53 (34.9%)	36 (37.5%)	17 (30.4%)	0.475
30‐day Reoperation	63 (41.4%)	34 (35.4%)	29 (51.8%)	0.071
Gastrointestinal Leak	19 (12.5%)	9 (9.4%)	10 (17.9%)	0.204
Graft Thrombosis	11 (7.2%)	10 (10.4%)	1 (1.8%)	0.098

*Note:* Data were mean ± standard deviation or number (percentage) for continuous or categorical variables, respectively.

Abbreviation: IFI, invasive fungal infection.

### 3.2 IFI

56 (36.8%) patients developed post‐transplant IFI. Forty‐one patients developed IFI with *Candida*, comprising six identified species (Table 2). The most common was *Candida glabrata* (recently renamed *Nakaseomyces glabratus*) (13 isolates, 31.7%), followed by *Candida albicans* (11 isolates, 26.8%) and *Candida parapsilosis* (9 isolates, 22.0%). *Candida krusei* (recently renamed Pichia kudriavzevii) accounted for 6 isolates (14.6%), while *Candida tropicalis* and *Candida kefyr* (formerly *C. pseudotropicalis*) were each represented by one isolate (2.4% combined). Overall, fluconazole resistance was identified in 12 of 41 isolates (29.3%), largely driven by the predominance of *Candida glabrata* isolates with elevated MICs and the presence of intrinsically fluconazole‐resistant *Candida krusei* . Resistance to other major antifungal classes was less frequent. Micafungin resistance was observed in 4 of 41 isolates (9.8%), primarily among *C. glabrata* . Amphotericin B resistance was rare, identified in 1 isolate (2.4%). Voriconazole generally retained activity against the majority of isolates, with only a small number demonstrating elevated minimum inhibitory concentrations. Detailed MIC values for amphotericin B, fluconazole, micafungin, and voriconazole are provided in Table S1. *Aspergillus* species were the second most common IFI, occurring in 9 patients (16.1%), and *Cryptococcus* species in 2 (3.6%). Less common etiologies included *Fusarium* (*n* = 1), *Mucorales* (*n* = 1), *Onychocola gallopava* (*n* = 1), and *Paecilomyces* (*n* = 1).

**TABLE 2:** Characteristics of invasive fungal infections among 56 intestine transplant recipients.

Organism (*n*, %)	Site(s) of infection (*n*, %)	Multisite infections (*n*)	30‐Day mortality
*Candida* species (41, 73.2%)	Bloodstream (10, 24.4%) IAI (31, 75.6%) Bloodstream + IAI (4, 9.8%) Bloodstream + Pulmonary (1, 2.4%)	5 (All were Blood + IAI or Blood + Pulmonary)	14 (34.1%)
*Aspergillus* species (9, 16.1%)	Pulmonary (8, 88.9%) Pulmonary + Skin (1, 11.1%)	1 (Pulmonary + Skin)	2 (22.2%)
*Cryptococcus* species (2, 3.6%)	CNS (1, 50.0%) Bloodstream + Skin (1, 50.0%)	1 (Blood + Skin)	0 (0.0%)
*Fusarium* species (1, 1.8%)	Bloodstream + Upper airway/palate (1, 1.8%)	1	0 (0.0%)
*Mucorales* (1, 1.8%)	Pulmonary (1, 100%)	0	0 (0.0%)
Other (2, 3.6%)	Pulmonary + CNS (*Onychocola gallopava*, 1, 50.0%) Skin (*Paecilomyces variotii*, 1, 50.0%)	*1 (Onychocola* case: Pulmonary + CNS)	0 (0.0%)
Total (56, 100%)	—	9 (16.1%)	16 (28.6%)

*Abbreviations:* CNS, central nervous system; IAI, intra‐abdominal infection; IFI, invasive fungal infection.

All patients received antifungal prophylaxis, most commonly with micafungin (*n* = 115, 75.7%). Antifungal prophylaxis was administered for a median of 21.0 days (IQR 14.0–36.3). Of patients with IFI, 21 (37.5%) developed breakthrough IFI despite currently receiving a prophylactic antifungal (17 micafungin and 4 amphotericin B lipid complex). Among 17 patients whose isolates had susceptibility data, 5 (29.4%) were caused by *Candida* isolates resistant to the utilised prophylactic antifungal agent, and 12 (70.6%) were susceptible.

Intra‐abdominal infection (*n* = 35, 62.5%) and bloodstream (*n* = 14, 25.0%) were the most frequent sites of infection, followed by pulmonary (*n* = 10, 17.9%), CNS (*n* = 3, 5.4%), skin (*n* = 2, 3.6%), and other sites (*n* = 3, 5.4%), which included lymph node, hard palate, and eye (Table 2). Most patients with IFI (*n* = 47, 83.9%) had infection confined to a single site, while 9 (16.1%) had multisite disease. Common site combinations included bloodstream and gastrointestinal tract (*Candida*, *n* = 4), bloodstream and pulmonary (*Candida*, *n* = 1), bloodstream and skin (*Cryptococcus*, *n* = 1), pulmonary and CNS (*Onychocola gallopava*, *n* = 1), bloodstream and palate (*Fusarium*, *n* = 1), and pulmonary and skin (*Aspergillus*, *n* = 1).

**FIGURE 1:**
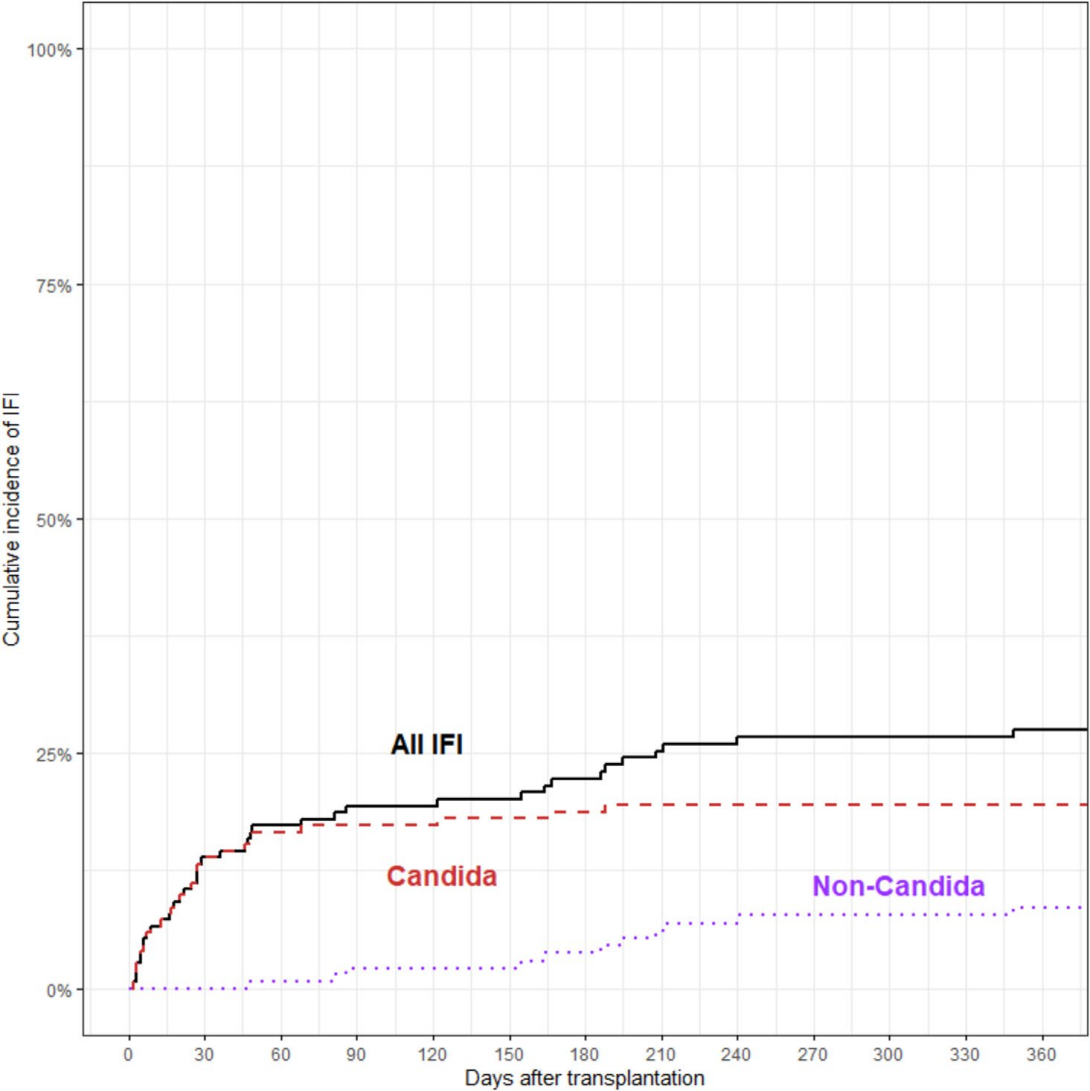
Kaplan–Meier curve comparing the incidence of invasive fungal infection (IFI) after transplantation. The black line indicates all IFIs, while the red, large‐dotted line represents *Candida* IFI and the purple, small‐dotted line represents non‐*Candida* IFI.

The median time to IFI onset was 83.5 days post‐transplant (IQR 19.5–444.2), with a range from 2 to 2,257 days. The timing of IFI after transplantation differed markedly among patients with *Candida* IFI and non‐*Candida* IFI (Figure 1). *Candida* IFIs occurred early, with a median time from transplant to *Candida* IFI of 29.0 days (IQR 13.0–469.0), reaching 19.5% cumulative incidence by one‐year post‐transplant. In contrast, non‐*Candida* IFIs were infrequent and occurred later, with a median time from transplant to non‐*Candida* IFI of 208.0 days (IQR 159.5–372.0) and a cumulative incidence of 8.5% at one‐year post‐transplant.

Initial antifungal therapy most commonly included micafungin (*n* = 31, 55.4%), an amphotericin B formulation (*n* = 16, 28.6%), and voriconazole (*n* = 12, 21.4%). Less frequently used agents were fluconazole (*n* = 4, 7.1%), posaconazole (*n* = 2, 3.6%), flucytosine (*n* = 1, 1.8%), and terbinafine (*n* = 1, 1.8%).

Among the 53 patients with pre‐transplant IFI, the majority had *Candida* infections (*N* = 49), followed by *Aspergillus* (*N* = 2), *Histoplasma* (*N* = 1), and other fungi (*N* = 1). Post‐transplant IFI occurred in 20 patients (37.7%), including 1 of 2 (50.0%) with pre‐transplant Aspergillus, 18 of 49 (36.7%) with Candida, and 1 of 1 (100.0%) with Histoplasma. Among patients with known post‐transplant IFI, the organisms most frequently identified were Candida (15/20, 75.0%), Aspergillus (4/20, 20.0%), and other fungi (1/20, 5.0%). Post‐transplant IFI occurred in 20/53 (37.7%) with pre‐transplant IFI and 36/98 (37%) without pre‐transplant IFI. There was no significant association between pre‐ and post‐transplant IFI (Fisher's exact test, *p* = 1.00).

To identify independent predictors of *Candida* IFI, we performed a multivariable Cox proportional hazards regression. After adjusting for potential confounders, redo transplantation (hazard ratio [HR] 2.73, 95% confidence interval [CI] 1.32–5.65; *p* = 0.007) and augmented immunosuppression (HR 3.25, 95% CI 1.30–8.11; *p* = 0.012) were significantly associated with an increased risk of *Candida* IFI. Alemtuzumab use was associated with a reduced risk of *Candida* IFI (HR 0.40, 95% CI 0.19–0.83; *p* = 0.014). Liver‐inclusive allograft showed a trend toward increased risk that did not reach statistical significance (HR 1.90, 95% CI 0.95–3.79; *p* = 0.069). Age at transplantation, anastomotic leak, and micafungin exposure were not significantly associated with *Candida* IFI (Table 3).

**TABLE 3:** Multivariable cox regression analysis for risk factors of candidiasis.

Variable	Hazard ratio	95% confidence interval	*p*‐value
Age at transplantation	1.01 per year	0.99–1.03	0.293
Alemtuzumab	0.40	0.19–0.83	0.014
Anastomotic leak	1.82	0.82–4.04	0.140
Micafungin prophylaxis	0.61	0.28–1.31	0.203
Redo transplantation	2.73	1.32–5.65	0.007
Augmented immunosuppression^a^	3.25	1.30–8.11	0.012
Liver‐inclusive allograft^b^	1.90	0.95–3.79	0.069

^a^ Analysed as a time‐dependent variable.

^b^ Includes liver‐intestine and full multivisceral allografts.

### 3.3 Death or Allograft Failure

Overall, 88 (57.9%) patients experienced death or allograft failure at a median time of 463.5 days (IQR 85.8–1467.2) post‐transplantation. This included 32 (21.1%) cases of allograft failure and 56 (36.8%) deaths with a functioning allograft; 38 (25.0%) of these events occurred within the first year. In a Cox proportional hazards model incorporating time‐dependent covariates, IFI was independently associated with an increased risk of death or allograft failure (HR 3.67, 95% CI 2.30–5.83; *p* < 0.001), after adjustment for age at transplantation, sex, anastomotic leak, graft thrombosis, redo transplantation, augmented immunosuppression, and diabetes mellitus. The median time from IFI diagnosis to death or allograft failure was 52.5 days (IQR 5.0–250.0). Notably, 16 patients died or experienced graft failure within 30 days of IFI diagnosis.

## 4 Discussion

IFI remains a major source of illness and death among intestinal transplant recipients [6]. Among solid organ transplant recipients, the 12‐month incidence of IFIs ranges from 1.3% to 11.6% [9]. In our cohort of 152 patients, we observed an incidence of 36.8%, reflecting the inherent vulnerabilities of this group, profound and prolonged immunosuppression, complex surgical procedures, and frequent reliance on central venous catheters and total parenteral nutrition. These findings are in line with published data, including the TRANSNET surveillance reports, which identify *Candida* species as the leading cause of IFIs in solid organ transplant recipients, with intestine allograft patients at the highest risk. Among non‐*albicans* species, *Candida glabrata* (recently renamed *Nakaseomyces glabratus*) represents the leading pathogen, comprising nearly 40% of all *Candida* isolates. While these infections are most frequently identified in patients undergoing liver or combined kidney‐liver transplantation, other species such as *Candida parapsilosis* (~6%) and *Candida krusei* (recently named *Pichia kudriavzevii*, ~5%) remain clinically significant. Notably, the emergence of these pathogens is often associated with prior antifungal exposure in solid organ transplant recipients [10, 11]. In our study, *Candida* accounted for 73.2% of all IFIs, with a shift toward non‐ *Candida. albicans* species. *Candida glabrata* was the most common isolate (31.7%), followed by *C. albicans* (26.8%) and *C. parapsilosis* (22.0%). This pattern is consistent with findings reported in other transplant populations, where shifts toward less susceptible species are frequently attributed to the selective pressure of azole prophylaxis and healthcare contact. In our cohort, however, echinocandins constituted the primary prophylactic strategy; thus, azole‐associated selection pressure alone is unlikely to account for the observed distribution. It is plausible that prior echinocandin exposure may similarly promote the emergence of isolates with elevated antifungal MICs, reflecting the broader principle that antifungal pressure can drive resistance development across drug classes [1, 12]. Notably, a substantial proportion of IFIs occurred despite antifungal prophylaxis, underscoring that in intestine transplant recipients, prophylaxis may not fully prevent infections arising from intra‐abdominal sources, particularly in the setting of mucosal barrier disruption and anastomotic complications.

In a multivariable analysis to identify risk factors for *Candida* IFI, redo transplantation and augmented immunosuppression emerged as independent risk factors for *Candida* IFI, underscoring the cumulative impact of host immune dysfunction and repeated allograft exposure. Interestingly, alemtuzumab use was associated with a reduced risk of *Candida* IFI. Although alemtuzumab is known to cause profound and prolonged lymphocyte depletion, this association may reflect confounding by indication, era effects, and broader changes in transplant practices over time, including shifts in antifungal prophylaxis and overall peri‐transplant management. In addition, patients receiving alemtuzumab may require less intensive maintenance immunosuppression and may experience lower rejection rates, which could indirectly reduce the risk of fungal translocation from the intestinal graft.

The majority of IFI events occurred early after transplantation, primarily due to *Candida* IFIs developing within the first 60–90 days. Similar to other reports, the pattern demonstrates that IFI risk in intestine transplant recipients is concentrated in the early postoperative period, coinciding with intense immunosuppression, mucosal barrier disruption, and postoperative complications [5, 13].

Intra‐abdominal was the most frequently involved IFI site (62.5%), consistent with its role as both a reservoir for colonisation and a portal for translocation of *Candida* species, especially in the setting of graft dysfunction or anastomotic leaks [5, 14]. Bloodstream infections were the next most common (25.0%), reflecting the heavy use of central venous catheters in this population. While most patients (83.9%) had infection confined to a single site, we also noted multisite disease, including rare cases such as *Onychocola gallopava* with combined CNS and pulmonary involvement. Notably, we did not identify any cases of *Pneumocystis jirovecii* pneumonia, likely reflecting the effectiveness of lifelong prophylaxis.

Importantly, IFIs were an independent predictor of death or allograft loss in the Cox analysis. While this highlights the clinical significance of IFIs in this population, these infections often occur in the setting of severe post‐transplant complications, and therefore may function both as contributors to adverse outcomes and as markers of underlying graft‐related complications and overall illness severity. Echinocandins were the most common empiric antifungal class used (55.4%), aligning with current guidelines recommending echinocandins as first‐line therapy for invasive candidiasis and candidemia due to their efficacy and safety profile [1]. High rates of fluconazole resistance in *C. glabrata* support the need for susceptibility testing and favour echinocandins or amphotericin B over azoles for empiric coverage in high‐risk patients [12, 14].

Interestingly, we found no association between pre‐ and post‐transplant IFIs (*p* = 1.00), suggesting that the post‐transplant environment marked by heavy immunosuppression, surgical stress, and invasive interventions plays a greater role than pre‐existing fungal colonisation in determining risk. However, knowledge of pre‐transplant IFI may have led to changes in antifungal prophylaxis, which may have mitigated risk as well. While mould infections such as *Aspergillus* (16.1%), *Fusarium*, and Mucorales were less common, which lines with reports from literature, and *Aspergillus fumigatus* is the most frequently isolated species regardless of the type of organ transplant [15], their presence underscores the need for broad antifungal vigilance. Rare and resistant fungi highlight the limitations of existing treatment options. Cryptococcosis accounts for approximately 8% of IFIs in solid organ transplant recipients, making it the third most prevalent fungal pathogen in this group. This infection occurs at a rate of 0.2% to 5% across all solid organ transplant types, with the highest frequency of cases documented in kidney transplant recipients and, less commonly, in liver transplant patients [16, 17]. Because the literature on IFIs in intestine transplantation is still sparse and often extrapolated from other solid organ transplants [1], larger multi‐centre studies are needed. Such research should aim to refine epidemiologic understanding, improve risk stratification, and assess emerging diagnostics and therapeutics to improve survival and quality of life for this highly vulnerable patient group.

This study is limited by its small sample size and retrospective design, which may introduce selection and information biases. Variability in diagnostic practices, immunosuppressive regimens, and prophylaxis over time may have influenced IFI detection and timing. We also included a relatively long study period, primarily due to the relative rarity of this transplant type, though changes in transplant practices over time may have affected outcomes. The low number of events also limits the ability to identify definitive risk factors for late‐onset infections. Additionally, we were unable to assess whether antifungal prophylaxis strategies were modified in patients with prior IFI, which may influence the interpretation of the observed lack of association between pre‐ and post‐transplant fungal infections.

In summary, IFI remains a significant complication after intestine transplantation and is closely associated with death or allograft loss. Our analysis highlights that multiple clinical factors, such as redo transplantation, gastrointestinal leak, and augmented immunosuppression, are key independent predictors of *Candida* IFI, likely reflecting the degree of immunosuppression and both medical and surgical complexity. These findings emphasise the need for vigilant long‐term monitoring, early recognition, and targeted preventive strategies to mitigate the impact of IFIs and improve post‐transplant outcomes in this high‐risk population.

## Author Contributions

**Blanca E. Gonzalez:** investigation, methodology, validation, writing – review and editing. **Jessica Lum:** methodology, writing – review and editing, validation, investigation. **Zachary A. Yetmar:** conceptualization, investigation, writing – review and editing, supervision, methodology, validation, visualization, formal analysis, data curation. **Mayyadah H. Alabdely:** conceptualization, investigation, writing – original draft, formal analysis, methodology, data curation. **Masato Fujiki:** methodology, investigation, validation, writing – review and editing.

## Funding

This study was supported by no funding was utilised in the preparation of this manuscript.

## Conflicts of Interest

The authors declare no conflicts of interest.

## Supporting information


**Table S1:** myc70180‐sup‐0001‐TableS1.docx. *Candida* Susceptibility Results.

## Data Availability

The data that support the findings of this study are available on request from the corresponding author. The data are not publicly available due to privacy or ethical restrictions.
