# Supplementary material for: Invasive Fungal Infections After Intestine Transplantation: Epidemiology and Outcomes
Source: Mycoses. 2026 Apr 28;69:e70180. doi: 10.1111/myc.70180 (PMC13122574; doi:10.1111/myc.70180)
Supplement: Supplementary file 1 — Table S1: myc70180‐sup‐0001‐TableS1.docx. Candida Susceptibility Results. [file MYC-69-e70180-s001.docx]

## Supplementary Table 1: *Candida* Susceptibility Results

| **Patient** | **Fungi** | **Amphotericin B** | **Fluconazole** | **Micafungin** | **Voriconazole** |
| --- | --- | --- | --- | --- | --- |
| 1 | *Candida albicans* | 0.5 | 0.25 | 0.015 |  |
| 2 | *Candida krusei* | 1.0 | 64 | 0.06 |  |
| 3 | *Candida krusei* | 0.5 | 32 | 0.12 |  |
| 4 | *Candida glabrata* | 0.5 | >256 | 0.06 |  |
| 5 | *Candida glabrata* | 0.5 | 8 | 2 |  |
| 6 | *Candida glabrata* | 0.5 | 16 | 0.05 |  |
| 7 | *Candida glabrata* | 1.0 | 8 | 0.02 |  |
| 8 | *Candida glabrata* | 0.5 | 64 | 4 |  |
| 9 | *Candida parapsilosis* | 1.0 | 0.5 | 2 |  |
| 10 | *Candida albicans* |  |  |  |  |
| 11 | *Candida glabrata* | 1.0 | 128 | 4 | 2.0 |
| 12 | *Candida glabrata* | 0.5 | 64 | 0.25 |  |
| 13 | *Candida albicans* |  |  |  |  |
| 14 | *Candida krusei* | 0.12 | 64 | 0.12 |  |
| 15 | *Candida krusei* | 1.0 | 64 | 0.12 |  |
| 16 | *Candida glabrata* | 1.0 | 32 | 1 | 2.0 |
| 17 | *Candida albicans* |  |  |  |  |
| 18 | *Candida kefyr (pseudotropicalis)* | 2.0 | 1 | 8 |  |
| 19 | *Candida krusei* | 0.5 | 32 | 0.12 |  |
| 20 | *Candida glabrata* | 1.0 | 16 | 0.015 |  |
| 21 | *Candida parapsilosis* |  |  |  |  |
| 22 | *Candida albicans* | 0.5 | 1 | 1 |  |
| 23 | *Candida albicans* | 0.5 | 1 | 0.015 |  |
| 24 | *Candida parapsilosis* | 0.25 | 1 | 1 |  |
| 25 | *Candida albicans* |  |  |  |  |
| 26 | *Candida glabrata* | 1.0 | 8 | 0.015 |  |
| 27 | *Candida parapsilosis* | 0.5 | 0.5 | 1 |  |
| 28 | *Candida krusei* | 2.0 | 128 | 0.25 |  |
| 29 | *Candida parapsilosis* |  |  |  |  |
| 30 | *Candida glabrata* | 1.0 | 8 | 0.015 |  |
| 31 | *Candida albicans* | 0.25 | 0.5 | 0.02 |  |
| 32 | *Candida parapsilosis* | 0.25 | 1 | 2 |  |
| 33 | *Candida albicans* | 0.5 | 0.5 | 2 |  |
| 34 | *Candida tropicalis* | 1.0 | 2 | 0.03 |  |
| 35 | *Candida glabrata* | 0.5 | 16 | 4 |  |
| 36 | *Candida parapsilosis* | 0.25 | 0.25 | 4 |  |
| 37 | *Candida parapsilosis* |  |  |  |  |
| 38 | *Candida glabrata* | 2.0 | 8 | 4 | 0.25 |
| 39 | *Candida albicans* |  |  |  |  |
| 40 | *Candida albicans* | 0.5 | 1 | ≤0.008 | 0.016 |
| 41 | *Candida parapsilosis* | 0.5 | 0.5 | 2 |  |

Blank cells indicate susceptibility testing for that antifungal was not performed.
